# Reliability of radiographic parameters in adenoid evaluation

**DOI:** 10.1590/S1808-86942012000400016

**Published:** 2015-10-20

**Authors:** Murilo Fernando Neuppmann Feres, Helder Inocêncio Paulo de Sousa, Sheila Márcia Francisco, Shirley Shizue Nagata Pignatari

**Affiliations:** 1 MSc (Doctoral student in the Otorhinolaryngology and Head and Neck Surgery Graduate Program of the University of São Paulo); 2 Orthodontist (private practice); 3 PhD (Professor and Head of the Pediatric Otorhinolaryngology Course at the Federal University of São Paulo). Universidade Federal de São Paulo

**Keywords:** adenoids, reproducibility of results, x-rays

## Abstract

The assessment of adenoids by x-ray imaging has been the topic of heated debate, but few studies have looked into the reliability of most existing radiographic parameters.

**Objective**: This study aims to verify the intra-examiner and inter-examiner reproducibility of the adenoid radiographic assessment methods.

**Materials and Methods**: This is a cross-sectional case series study. Forty children of both genders aged between 4 and 14 were enrolled. They were selected based on complaints of nasal obstruction or mouth breathing and suspicion of pharyngeal tonsil hypertrophy. Cavum x-rays and orthodontic teleradiographs were assessed by two examiners in quantitative and categorical terms.

**Results**: All quantitative parameters in both x-ray modes showed excellent intra and inter-examiner reproducibility. Relatively better performance was observed in categorical parameters used in cavum x-ray assessment by C-Kurien, C-Wang, C-Fujioka, and C-Elwany over C-Cohen and C-Ysunza. As for orthodontic teleradiograph grading systems, C-McNamara has been proven to be more reliable than C-Holmberg.

**Conclusion**: Most instruments showed adequate reproducibility levels. However, more research is needed to properly determine the accuracy and viability of each method.

## INTRODUCTION

The assessment of pharyngeal tonsil hypertrophy by lateral x-ray images of the skull has been the target of debate for years^1^, ^2^, ^3^, ^4^. Nevertheless, opinions on the usefulness of these images still vary significantly.

These differences of opinions are, among other factors, the outcome of the lack of studies simultaneously looking into a considerable number of parameters, of the diversity seen in the studied samples, and of the application of various methods, some of which questionable^5^. Among these shortcomings is the frequent absence of reliability tests for most radiographic parameters^5^.

Reproducibility is an essential requirement to determine the quality of any assessment parameter. Therefore, this study was developed with the purpose of verifying the intra and inter-examiner reproducibility of a series radiographic parameters used to assess the pharyngeal tonsil and the nasal pharyngeal airway.

## MATERIALS AND METHODS

This cross-sectional study was approved by the Research Ethics Committee of the institution in which it was carried out and given permit n^o^ 0181/08).

### The sample

Forty children (n = 40) of both genders with ages ranging between 4 and 14 years were selected at the Pediatric ENT Ward of the institution in which the study was carried out. The enrolled patients shared complaints of nasal obstruction and/or mouth breathing, and were suspected for pharyngeal tonsil hypertrophy. Syndromic children, patients with malformations, individuals with acute respiratory tract infection at the time of examination, and subjects with a history of adenoidectomy were excluded. The guardians of the children enrolled in the study formalized their participation by signing an informed consent term as per the requirements of the Research Ethics Committee of the institution in which the study was carried out.

### Methods

#### Cavum x-rays

One radiologist took cavum x-rays of the selected children at a specialized center. All x-ray images were made on the same apparatus at a focus-film distance of 140 cm and exposure factors of 70 kV, 12 mA for 0.40 to 0.64 seconds. Patients were positioned in a standing position in a way that the horizontal plane of Frankfurt was parallel to the floor and the central beam of x-rays were directed to the nasopharynx. The children were advised to breathe through their noses keeping their mouths closed and teeth occluded as x-ray images were taken. x-ray film used was Kodak® 20 cm × 25 cm which after exposure was developed automatically according to the standard method. Images showing elevated soft palates or significant rotation of the head were discarded and the respective subjects removed from the sample.

#### Lateral orthodontic teleradiography (TR)

TR images were captured by the same operator. The same exposure, patient positioning, and patient orientation used in cavum x-rays were used in TR. This turn, however, a device called cephalostat was used to ensure proper reproducible patient head positioning as x-ray images were produced. The central x-ray beam was directed towards the external acoustic meatus. Film, development method, and other exclusion criteria were the same as used in cavum x-rays.

**Figure 1:**
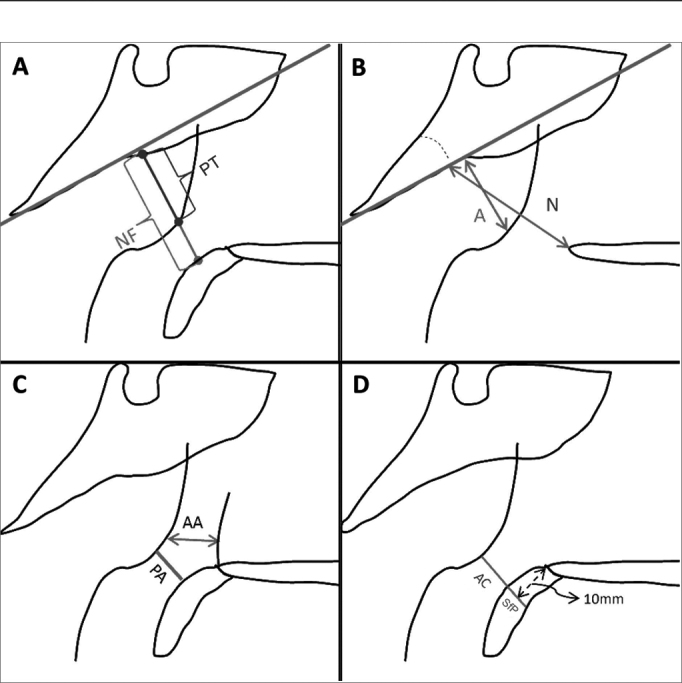
Cavum x-ray parameters. (A): PT: pharyngeal tonsil; NF: nasopharynx. (B): A: adenoid; N: nasopharynx. (C): AA: antral-adenoid; PA: passage of air. (D): AC: air column; SfP: soft palate.

**Figure 2:**
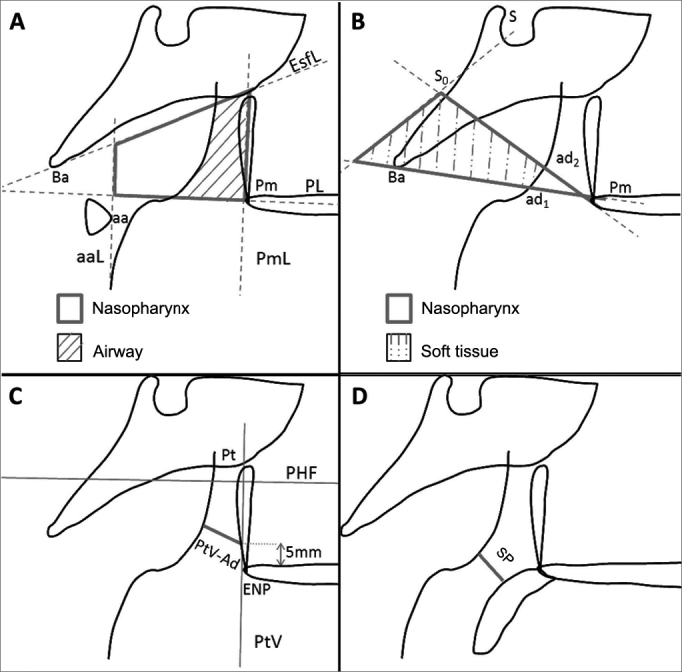
Cavum x-ray parameters. (A): Ba: basion (the most inferior point over the anterior border of the foramen magnum); EsfL: sphenoid line (tangent to the inferior border of the sphenoid bone in relation to Ba); PL: palate line (from the anterior to the posterior nasal spine); Pm: pterygomaxillary (the intersection between the border of the nasal floor and the posterior border of the maxilla); PmL: pterygomaxillary line (perpendicular to PL in relation to Pm); aa: anterior atlas (most anterior point of the atlas); aaL: anterior atlas line (perpendicular to PL in relation to aa). (B): S: sella (situated in the geometric center of the sella turcica); Ba: basion; S_0_: mid-point in the distance between S-Ba; Pm: pterygomaxillary; ad_1_: intersection between line Pm-Ba and the border of the pharyngeal tonsil; ad_2_: intersection between line Pm-S_0_ and the border of the pharyngeal tonsil. (C): PHF: Frankfurt horizontal plane; Pt: pterygoid (point located in the intersection between the inferior border of the round foramen and the posterior portion of the pterygopalatine fossa); PtV: vertical pterygoid (line perpendicular to PHF in relation to Pt); PtV-Ad: distance between the border of the pharyngeal tonsil and PtV. (D): SP: superior pharynx.

Each radiographic image (cavum x-rays and TR) was given a number to mask patient and to prevent examiners from knowing the subjects' respiratory symptoms and initial complaints. Two independent examiners looked at the tracings of anatomic structures and assessed the images. The independent examiners were not involved in patient enrollment or patient examination. The main examiner (Examiner 1) performed radiographic measurements (Charts 1 and 2; Figures 1 and 2) twice at different times with a 30-day interval between them, to allow for truly independent assessment.

**Chart 1:** Cavum x-ray assessment methods and their respective references.

Reference Study	Assessment Method
Jóhannesson^8^	Pharyngeal tonsil thickness (PT) (mm): distance measured along a perpendicular line until the superior bone border of the nasopharynx from the pharyngeal tubercle to the convexity of the pharyngeal tonsil (Figure 1A).
Fujioka et al.^9^	Adenoid/Nasopharynx ratio (A/N): ratio between the thicknesses of the adenoid (A) and the nasopharynx (N), being A the distance along a line perpendicular to the straight portion of the anterior border of the basioccipital bone and the point of greatest convexity in the pharyngeal tonsil; and N as the distance between the posterior and superior portion of the hard palate and the anterior border of the spheno-occipital synchondrosis (Figure 1B).
	Pharyngeal tonsil categories (C-Fujioka): “Normal” (A/N ≤ 0.8), “Enlarged” (A/N > 0.8).
Crepeau et al.^10^	Antral adenoid (AA) (mm): shortest distance between the most anterior portion of the pharyngeal border and the posterior wall of the maxillary antrum located on the same plane as the choana (Figure 1C).
Maw et al.^11^	Passage of air (PA) (mm): shortest distance between the pharyngeal tonsil convexity and soft palate (Figure 1C).
	Air column (AC) (mm): distance between the posterior border of the soft palate 10 mm away from the posterior nasal spine and the anterior curvature of the pharyngeal tonsil border (Figure 1D).
Cohen & Konak^12^	Air column/soft palate ratio (AC/SfP): ratio between AC (see description above) and SfP, the latter being the thickness of the soft palate measured 10 mm away from the posterior nasal spine (Figure 1D).
	Pharyngeal tonsil categories (C-Cohen): “Small” (AC/SfP ≥ 1.0), “Medium” (0.5 ≤ AC/SfP < 1.0), “Large” (AC/SfP < 0.5).
Elwany^13^	Pharyngeal tonsil categories (C-Elwany): “Normal” (A/N ≤ 0.7), “Enlarged” (A/N > 0.73).
Wang et al.^1^	Subjective categorization of pharyngeal tonsil hypertrophy (C-Wang): “Not obvious”, “Obvious”.
Mlynarek et al.^2^	Airway occlusion (AWO) (%): percent relationship between PT (see description above) and NF, the latter being the distance measured along a line perpendicular to the superior bone border of the nasopharynx from the pharyngeal tubercle to the soft palate. (Figure 1A).
Kurien et al.^3^	Categorization of pharyngeal tonsil hypertrophy (C-Kurien): “Grade 1” (PA ≥ 6.0 mm), “Grade 2” (3.0 mm ≤ PA < 6.0 mm), “Grade 3” (PA < 3.0 mm).
Ysunza et al.^4^	Subjective categorization of pharyngeal tonsil hypertrophy (C-Ysunza): “Grade 1”, “Grade 2”, “Grade 3”, “Grade 4”.

**Chart 2:** Teleradiography assessment methods and their respective references.

Reference Study	Assessment Method
Handelman & Osborne^6^	Nasopharyngeal airway area (Npaa) (%): (Figure 2A).
Schulhof^14^	PtV-Ad (mm): the shortest distance between the adenoid border and the PtV (5mm above the posterior nasal spine nasal posterior) (Figure 2C).
Holmberg & Linder-Aronson^15^	Subjective categorization of pharyngeal tonsil (C-Holmberg): “Small”, “Moderate”, “Large”, “Very Large”.
	Sagittal depth (1) of the airway (Pm-ad_1_) (mm) (Figure 2B).
	Sagittal depth (2) of the airway (Pm-ad_2_) (mm) (Figure 2B).
Linder-Aronson & Leighton^7^	Soft tissue thickness (1) (ad_1_-Ba) (mm) (Figure 2B).
	Soft tissue thickness (2) (ad_2_-S_0_) (mm) (Figure 1B).
	Soft tissue area (Ad/Nf) (%): (Figure 2B).
	Sagittal depth of the osseous nasopharynx (Pm-Ba) (mm) (Figure 2B).
McNamara Jr.^16^	Superior pharynx (SP) (mm): shortest distance from a point on the superior border of the soft palate and a point on the border of the pharyngeal tonsil (Figure 1D).
	Airway categorization (C-McNamara): “Non obstructive” (SP > 5), “Apparently obstructive” (SP ≤ 5).

Tracings and further measurements were made on Ultraphan paper towels with the aid of a negastocope, ruler, square, and a Starret™ (model 799A- 8/200) digital caliper with 0.01 mm divisions. Area calculations (*Npaa^6^); (Ad/Nf*^7^) were carried out with the aid of software program ImageJ available for download at http://rsbweb.nih.gov/ij/download.html after the cephalometric tracings had been scanned.

### Analysis methods

The reliability of radiographic methods was determined by the analysis of intra and inter-examiner reproducibility. Reproducibility of quantitative radiographic variables was measured in terms of the interclass correlation coefficient (ICC) and the mean differences between pairs of observations. Reliability analysis of categorical radiographic variables was performed by calculating the kappa (k) coefficient and the overall agreement percentage between paired observations, including the occurrence of random agreement. ICC was interpreted according to Weir et al.^17^, wherein reliability was categorized as “low” (CCI ≤ 0.20), “fair” (0.20 < CCI ≤ 0.40), “good” (0.40 < CCI ≤ 0.60), “very good” (0.60 < CCI ≤ 0.80) or “excellent” (0.80 < CCI ≤ 1.00). The value of the kappa coefficient was interpreted based on the criteria designed by Landis & Koch^18^, in which reliability was rated “low” (k ≤ 0.20), “fair” (0.20 < k ≤ 0.40), “moderate” (0.40 < k ≤ 0.60), “substantial” (0.60 < k ≤ 0.80) or “nearly perfect” (0.80 < k ≤ 1.00).

The level of statistical significance established for statistical tests was 5% (α≤ 0.05). Statistical analysis was done using software program SPSS 10.0 for Windows.

## RESULTS

Eleven patients refused to participate in the study. One patient was excluded for inconclusive x-ray images.

Forty subjects were enrolled in this study, twenty (50.0%) females and twenty (50.0%) males. Patient mean age was 9.5 years (4.1-14.3 years; standard deviation of 2.4 years). All included patients were suspected for pharyngeal tonsil hypertrophy (40/40, 100.0%). Most of them complained of mixed breathing (19/40; 47.5%) or mouth breathing alone (17/40; 42.5%).

Every cavum x-ray (Table 1) and teleradiography (Table 2) quantitative parameter was rated as excellent for both intra and inter-examiner reproducibility.

**Table 1:** Interclass correlation coefficient (ICC) of the quantitative cavum x-ray parameters in relation to the first and second measurements done by Examiner 1 (intra-examiner analysis) and to the measurements done by examiners 1 and 2 (inter-examiner analysis).

	Intra-examiner		Inter-examiner	
Variables	ICC	*p*	ICC	*p*
PT (mm)	0.969	< 0.001	0.920	< 0.001
A/N	0.952	< 0.001	0.942	< 0.001
AA (mm)	0.975	< 0.001	0.942	< 0.001
PA (mm)	0.985	< 0.001	0.972	< 0.001
AC (mm)	0.964	< 0.001	0.940	< 0.001
AC/SfP	0.928	< 0.001	0.850	< 0.001
AWO (%)	0.957	< 0.001	0.936	< 0.001

**Table 2:** Interclass correlation coefficient (ICC) of the quantitative teleradiography parameters in relation to the first and second measurements done by Examiner 1 (intra-examiner analysis) and to the measurements done by examiners 1 and 2 (inter-examiner analysis).

	Intra-examiner		Inter-examiner	
Variables	ICC	*P*	ICC	*P*
Npaa (%)	0.97	< 0.001	0.91	< 0.001
PtV-AD (mm)	0.98	< 0.001	0.94	< 0.001
Pm-ad_1_ (mm)	0.99	< 0.001	0.98	< 0.001
Pm-ad_2_ (mm)	0.98	< 0.001	0.96	< 0.001
ad_1_-Ba (mm)	0.99	< 0.001	0.95	< 0.001
ad_2_-S_0_ (mm)	0.98	< 0.001	0.96	< 0.001
Pm-Ba (mm)	0.97	< 0.001	0.89	< 0.001
Ad/Nf (%)	0.97	< 0.001	0.95	< 0.001
SP (mm)	0.98	< 0.001	0.96	< 0.001

Clinically insignificant variations were also observed when comparing measurements done by the same examiner in two occasions or by two examiners (Table 1, Table 2).

**Table 3:** Differences between paired observations for quantitative cavum x-ray parameters in relation to the first and second measurements done by Examiner 1 (intra-examiner analysis) and to the measurements done by examiners 1 and 2 (inter-examiner analysis).

		Intra-examiner				Inter-examiner		
Variables	Mean^a^	SD^b^	Min^c^	Max^d^	Mean^a^	SD^b^	Min^c^	Max^d^
PT (mm)	0.6483	0.5162	-2.4500	0.7000	0.9345	0.9233	-3.9200	1.5400
A/N	0.0289	0.0241	-0.0541	0.1117	0.0294	0.0267	-0.1065	0.0984
AA (mm)	0.4383	0.3706	-0.9600	1.5000	0.6828	0.5052	-2.0900	1.200
PA (mm)	0.3960	0.3104	-0.8800	1.1300	0.5100	0.4638	-1.5900	2.100
AC (mm)	0.5843	0.5780	-2.7600	1.5800	0.8415	0.6517	-2.5200	2.6400
AC/SfP	0.1190	0.1095	-0.3308	0.5421	0.1690	0.1440	-0.4396	0.5490
AWO (%)	2.7170	2.1017	-7.5850	3.6105	3.1871	2.7210	-9.3155	11.3128

a considering the absolute differences between paired observations;

b standard deviation

c minimum value

d maximum value.

**Table 4:** Differences between paired observations for quantitative teleradiography parameters in relation to the first and second measurements done by Examiner 1 (intra-examiner analysis) and to the measurements done by examiners 1 and 2 (inter-examiner analysis).

		Intra-examiner				Inter-examiner		
Variables	Mean^a^	SD^b^	Min^c^	Max^d^	Mean^a^	SD^b^	Min^c^	Max^d^
Npaa (%)	1.82	1.84	-10.04	4.65	3.35	2.92	-13.31	6.96
PtV-AD (mm)	0.43	0.33	-1.23	1.20	0.78	0.67	-2.91	1.21
Pm-ad_1_ (mm)	0.39	0.29	-0.99	1.01	0.66	0.52	-2.38	2.20
Pm-ad_2_ (mm)	0.40	0.27	-1.00	1.12	0.67	0.58	-3.23	1.46
ad_1_-Ba (mm)	0.60	0.40	-1.53	1.78	1.17	0.91	-3.57	1.59
ad_2_-S_0_ (mm)	0.41	0.30	-1.33	1.04	0.73	0.56	-2.28	1.88
Pm-Ba (mm)	0.57	0.45	-1.73	1.98	1.20	0.84	-3.99	1.45
Ad/Nf (%)	1.18	0.86	-2.30	3.61	1.30	1.11	-3.99	4.84
UP (mm)	0.34	0.42	-2.63	0.66	0.45	0.61	-3.58	1.21

a considering the absolute differences between paired observations

b standard deviation

c minimum value

d maximum value.

In cavum x-ray categorical variables, *C-Kurien* had “nearly perfect” agreement in intra and inter-examiner analysis. Great agreement percentages were also found in intra (90.0%) and inter-examiner (92.5%) analysis (Table 5).

**Table 5:** Kappa (k) coefficient of categorical cavum x-ray parameters in relation to the first and second measurements done by Examiner 1 (intra-examiner analysis) and to the measurements done by examiners 1 and 2 (inter-examiner analysis).

Intra-examiner					
C-Fujioka					
	2^nd^ observation				
1^st^ observation	Normal	Enlarged	Total	k	*p*
	**35**	_-_	35		
Normal	**87.5%**	-	87.5%	0.724	< 0.001
Enlarged	2	**3**	5		
	5.0%	**7.5%**	12.5%		
	37	3	40		
Total	92.5%	7.5%	100.0%		
C-Elwany					
	2^nd^ observation				
1^st^ observation	Normal	Enlarged	Total	k	^*P*^
	**29**	3	32		
Normal	**72.5%**	7.5%	80%	0.714	< 0.001
Enlarged	1	**7**	820%		
	2.5%	**17.5%**			
	30	10	40		
Total	75%	25%	100%		

C-Cohen						
	2^nd^ observation					
1^st^ observation	Small	Medium	Large	Total	k	^*P*^
	**19**	2	_-_	21		
Small						
	**47.5%**	5%	-	52.5%		
	2	**10**	3	15		
Medium					0.564	< 0.001
	5%	**25%**	7.5%	37.5%		
	_-_	3	**1**	4		
Large						
	-	7.5%	**2.5%**	10%		
	21	15	4	40		
Total	52.5%	37.5%	10%	100%		

C-Wang					
	2^nd^ observation				
1^st^ observation	Not obvious	Obvious	Total	k	*p*
	**23**	2	25		
Not obvious	**57.5%**	5%	62.5%	0.896	< 0.001
	_-_	**15**	15		
Obvious	-	**37.5%**	37.5%		
	23	17	40		
Total	57.5%	42.5%	100%		

C-Kurien						
	2^nd^ observation					
1^st^ observation	Grade 1	Grade 2	Grade 3	Total	k	*p*
	**25**	_-_	_-_	25		
Grade 1					0.807	< 0.001
	**62.5%**	-	-	62.5%		
	1	**8**	2	11		
Grade 2	2.5%	**20%**	5%	27.5%	0.807	< 0.001
	_-_	1	**3**	4		
Grade 3	-	2.5%	**7.5%**	10%		
	26	9	5	40		
Total	65%	22.5%	12.5%	100%		

C-Ysunza							
		2^nd^ observation					
1^st^ observation	Grade 1	Grade 2	Grade 3	Grade 4	Total	k	*p*
	**8**	7	_-_	_-_	15		
Grade 1	**20%**	17.5%	-	-	37.5%		
	_-_	**8**	3	1	12		
Grade 2	-	**20%**	7.5%	2.5%	30%	0.525	< 0.001
	_-_	1	**6**	2	9		
Grade 3	-	2.5%	**15%**	5%	22.5%		
	_-_	_-_		**4**	4		
Grade 4	-	-		**10%**	10%		
	8	16	9	7	40		
Total	20%	40%	22.5%	17.5%	100%		

Inter-examiner					
C-Fujioka					
	Examiner 2				
Examiner 1	Normal	Enlarged	Total	k	*p*
	**35**	_-_	35		
Normal	**87.5%**	-	87.5%	0.724	< 0.001
Enlarged	2	**3**	5		
	5%	**7.5%**	12.5%		
	37	3	40		
Total	92.5%	7.5%	100%		
C-Elwany					
	Examiner 2				
Examiner 1	Normal	Enlarged	Total	k	*p*
	**30**	2	32		
Normal	**75%**	5%	80%	0.776	< 0.001
Enlarged	1	**7**	8		
	2.5%	**17.5%**	20%		
	31	9	40		
Total	77,5%	22,5%	100%		

C-Cohen						
	Examiner 2					
Examiner 1	Small	Medium	Large	Total	k	*p*
	**17**	4	_-_	21		
Small	**42.5%**	10%	-	52.5%	0.562	< 0.001
	3	**11**	1	15		
Medium	7.5%	**27.5%**	2.5%	37.5%		
Large	-	2	**2**	4	0.562	< 0.001
	-	5%	**5%**	10%		
	20	17	3	40		
Total	50%	42.5%	7.5%	100%		

C-Wang					
	Examiner 2				
Examiner 1	Not obvious	Obvious	Total	k	*p*
	**22**	3	25		
Not obvious	**55%**	7.5%	62.5%	0.792	< 0.001
	1	**14**	15		
Obvious	2.5%	**35%**	37.5%		
	23	17	40		
Total	57.5%	42.5%	100%		

C-Kurien						
	Examiner 2					
Examiner 1	Grade 1	Grade 2	Grade 3	Total	k	*p*
	**23**	2	_-_	25		
Grade 1	**57.5%**	5%	-	62.5%		
	1	**10**	_-_	11		
Grade 2	2.5%	**25%**	-	27.5%	0.859	< 0.001
	_-_	_-_	**4**	4		
Grade 3	-	-	**10%**	10%		
	24	12	4	40		
Total	60%	30%	10%	100%		

C-Ysunza							
		Examiner 2					
Examiner 1	Grade 1	Grade 2	Grade 3	Grade 4	Total	k	*p*
	**5**	9	1	_-_	15		
Grade 1	**12.5%**	22.5%	2.5%	-	37.5%		
	2	**4**	6	_-_	12		
Grade 2	5%	**10%**	15%	-	30%	0.207	0.025
	_-_	3	**6**	_-_	9		
Grade 3	-	7.5%	**15%**	-	22.5%		
	_-_	_-_	2	**2**	4		
Grade 4	-	-	5%	**5%**	10%		
	7	16	15	2	40		
Total	17.5%	40%	37.5%	5%	100%		

Agreements in bold type.

*C-Wang* had “nearly perfect” agreement levels in intra-examiner agreement and “substantial” agreement in inter-examiner analysis. Agreement percentages were 95.0% and 90.0% respectively (Table 5).

*C-Fujoka* and *C-Elwany* had “substantial” kappa agreement for both analyses. Different measurements (*C-Fujioka*: 95.0%; *C-Elwany*: 90.0%) or examiners (*C-Fujioka*: 95.0%; *C-Elwany*: 92.5%) had agreement in a significant portion of the assessments (Table 5).

*C-Cohen* had “moderate” performance based on the obtained kappa indices. Agreement rates mounted to 75.0% for both intra and inter-examiner analyses (Table 5).

Additionally to “moderate” agreement in the intra-examiner analysis, *C-Ysunza* was rater “fair” when looking at different examiners. Percentages of correct answers were 65.0% on intra-examiner analysis and 42.5% on inter-examiner analysis (Table 5).

*C-McNamara* had “nearly perfect” agreement in the kappa coefficient for intra and inter-examiner performance (Table 6). The rate of agreement was 97.5% between observations and 95.0% between different examiners.

**Table 6:** Kappa (k) coefficient of categorical teleradiography parameters in relation to the first and second measurements done by Examiner 1 (intra-examiner analysis) and to the measurements done by examiners 1 and 2 (inter-examiner analysis)

Intra-examiner							
C-Holmberg							
		2^nd^ observation					
1^st^ observation	Small	Mod^a^	Large	VL^b^	Total	k	*p*
	**4**	3	_-_	_-_	7		
Small						0.673	< 0.001
	**10.0%**	7.5%	-	-	17.5%		
	_-_	**18**	2	_-_	20		
Mod^a^	-	**45.0%**	5.0%	-	50.0%		
Large	-	2	**9**	**1**	12	0.673	< 0.001
	-	5.0%	**22.5%**	**2.5%**	30.0%		
	_-_	_-_	_-_	1	1		
VL^b^	-	-	-	2.5%	2.5%		
	4	23	11	2	40		
Total	10.0%	57.5%	27.5%	5.0%	100.0%		

C-McNamara					
	2^nd^ observation				
1^st^ observation	Non obstructive	Apparently obstructive	Total	k	*p*
	**28**	_-_	28		
Non obstructive	**70.0%**	-	70.0%	0.939	< 0.001
Apparently obstructive	1	**11**	12		
	2.5%	**27.5%**	30%		
	29	11	40		
Total	72.5%	27.5%	100%		

Inter-examiner							
C-Holmberg							
		Examiner 2					
Examiner 1	Small	Mod^a^	Large	VL^b^	Total	k	^*P*^
	**7**	_-_	_-_	_-_	7		
Small	**17.5%**				17.5%		
	12	**8**	_-_	_-_	20		
Mod^a^	30%	**20%**	-	-	50%	0.414	< 0.001
Large	-	3	**7**	2	12		
	-	7.5%	**17.5%**	5%	30%		
	_-_	_-_	_-_	**1**	1		
VL^b^	-	-	-	**2.5%**	2.5%		
	19	11	7	3	40		
Total	47.5%	27.5%	17.5%	7.5%	100%		

C-McNamara					
	Examiner 2				
Examiner 1	Non obstructive	Apparently obstructive	Total	k	*p*
	**28**	_-_	28		
Non obstructive	**70%**	-	70%	0.875	< 0.001
Apparently obstructive	2	**10**	12		
	5%	**25%**	30%		
	30	10	40		
Total	75%	25%	100%		

a moderate;

b ^b^very large; agreements in bold type.

*C-Holmberg* had “substantial” agreement in intra-examiner performance and “moderate” agreement for inter-examiner performance (Table 6). This parameter had the following agreement percentages – intra-examiner: 80.0%; inter-examiner: 57.5%.

## DISCUSSION

### Cavum x-rays

Quantitative variables had excellent reproducibility among examiners. Previous studies reported similar results for *AN*^13^, ^19^, *PA*^19^ e *AA*^19^. Other quantitative parameters (*PT, AC, AC/SfP, AWO)*, although not investigated previously, were also in agreement with the data of this study and presented excellent inter-examiner reliability. The results for intra-examiner performance seen in this study showed for the first time excellent rates of reproducibility for all investigated instruments. Therefore, quantitative parameters may be reliably used researchers and physicians specialized in this area.

However, less consistency was observed in relation to categorical cavum x-ray variables. In this case, various reproducibility rates were observed, ranging from fair to nearly perfect.

Instrument *C-Kurien* outperformed all other tested categorization systems. The excellent rates of reproducibility connected to the presence of reliable objective categorization criteria (*PA*) grant this instrument outstanding levels of reliability.

*C-Wang* also had satisfactory levels of reproducibility, even when submitted to the subjective impressions of examiners. Its performance may be related to the fact that examiners tend to systematically categorize doubtful cases as “non-obvious” hypertrophy. Therefore, albeit reliable, this assessment instrument should be used carefully by examiners.

Satisfactory levels of reproducibility were also observed for *C-Fujioka* and *C-Elwany*, whose categorization criteria are based on the A/N value. These instruments should be used in cases in which the characterization of the nasopharyngeal airway needs to be done in a simplified (dichotomic categories) and objective manner.

Despite the moderate levels of intra-examiner reliability, *C-Cohen* was rated as a reproducible system by Souki^20^. Kolo et al.^21^ as high agreement rates were reported between an ENT and a radiologist (k = 0.8182; agreement rate of 82.35%). However, when agreement was verified between two ENT physicians, more modest performance was observed (k = 0.6696; agreement rate of 74.51%)^21^, and closer to the reproducibility rates observed in our study.

Lower levels of performance on categorization parameters was observed in instrument *C-Ysunza*. Other studies reported inter-examiner agreement rates ranging between 77.5%^11^ and 90.0% of the assessments^4^; agreement rates seen in our study were lower. According to Maw et al.^11^, this type of assessment is highly dependent on examiner experience; the assessments on Ysunza et al.^4^ were performed by experienced personnel. This instrument requires experienced examiners. Therefore, training is needed before the *C-Ysunza* instrument is used, despite the substantial levels of agreement seen in intra-examiner analysis.

### Teleradiography

According to the data collected, all investigated quantitative parameters had excellent intra-examiner reproducibility. These findings are in agreement with other studies^20^, ^22^, ^23^, ^24^ in which statistically significant intra-examiner variations and clinically insignificant differences were found. Although the literature on orthodontics has found parameters *Npaa*^20^, *Pm-ad_1_*^21^, ^24^, *Pm-ad_2_*^22^, ^24^, ad_1_-Ba^22^, ^24^, *ad_2_-S_0_*^22^, ^23^, *Pm-Ba*^22^, ^24^, e *SP*^20^, ^24^ to have satisfactory intra-examiner reliability, other variables such as *PtV-Ad* and *Ad/NP* were also proven to offer sufficient intra-examiner reproducibility.

No studies in the literature have verified the inter-examiner reproducibility of these radiological variables. However, the results of this study suggest they offer satisfactory agreement between examiners. Our findings have confirmed the reliability of quantitative methods, and their appropriateness for practical use.

When looking at the reproducibility of categorization systems, this study found excellent agreement rates intra and inter-examiners using *C-McNamara*. However, *C-Holmberg* - a system based on subjective examiner impressions - was not as well rated as *C-McNamara*, specifically on inter-examiner reproducibility.

Paradise et al.^25^, using a categorization system similar to *C-Holmberg*, found excellent rates of reproducibility (intra-examiner: k = 0.89; inter-examiner: k = 0.81). Souki et al.^20^ studied the intra-examiner reproducibility rates for the same parameter and did not find statistically significant differences between the intra-examiner paired mean values. Our study also revealed a considerable agreement rate for intra-examiner analyses. Even so, the authors of this study recommend that *C-McNamara* be given preference. The absence of defined criteria and objectives in *C-Holmberg*, the excessive number of categories, and the lower rates of inter-examiner agreement should be enough justification to use *C-McNamara*, a simpler, more objective and more reliable categorization system.

Other requirements than reproducibility should be considered when picking a diagnostic method, such as viability and accuracy. That is why further research is required to determine the capacity each parameter analyzed in this study has to represent what they are intended for. The ideal instrument should be reliable, accurate, and practical.

## CONCLUSION

Every quantitative parameter measured on cavum x-rays or teleradiography presented excellent reproducibility and clinically irrelevant variation.

The top performers among the categorical parameters observed in cavum x-rays were *C-Kurien*, *C-Wang, C-Fujioka* and *C-Elwany* over *C-Cohen* and *C-Ysunza*.

*C-McNamara* outperformed *C-Holmberg* in reproducibility among teleradiography-based categorization systems.
